# The Sufferings of Christ

**Published:** 1888-04-14

**Authors:** 


### WORDS OF CONSOLATION.

[Communications for this column should be addressed "Chaplain," care of the Editor of The Hospital, who will gratefully welcome any suggestions or inquiries from matrons, nurses, and the staff generally.]

### LXXIII.—The Sufferings of Christ.

*For Reading to the Sick.*

We are now endeavouring to show you that the idea of our Blessed Lord's sacrifice on the Cross alone being the pledge and security of individual and actual salvation is a mistaken one. That which took place for us more than eighteen hundred years ago will not save us unless we become individually partakers of the fruits of the sacrifice. The sacrifice on Calvary made salvation attainable for all. So Christ died for all, in so far as that He has made salvation possible for all. But salvation has also to be conferred on individual souls. They have to be personally brought into contact with their Redeemer. Christ is our Redeemer in two senses. He has died for us. But if we would be saved, He has something to do in us as well. The constant burden of His discourses in St. John's Gospel is evidently to show that He would surrender His life in order to return it the more effectually to His people. There is the oft-repeated promise of the Holy Spirit Whom He would send to dwell within His disciples. There is the promise of His own flesh and blood, which He would give for the life of the world. If you read St. John attentively, you will notice that, in nearly every case of a promise being made of these spiritual gifts, all depends upon His first dying, rising, and ascending to the Father. The key almost to the entire discourse uttered after the institution of the Lord's Supper is that a great change must pass upon Him through death, in order that He might return to His own the more effectually. (See John xiv.-xvii.) Now probably the common and popular way of speaking of the precious blood of Christ, or of the shedding of His blood, may be Scriptural so far as it goes. Nevertheless, some of our Bible and Church teaching would be much more intelligible, and people would have more definite ideas about redemption and sanctification were it remembered that the reason for Christ's Life being outpoured is, we repeat, that the benefits of the Atonement may be transferred to us individually. This point needs insisting upon, because numbers of people rest their hopes of salvation entirely upon the propitiatory and expiatory part of the Atonement, without seeing that such faith alone is insufficient. They need to realize how the corn of wheat died, in order that they might partake of the fruit which springs from it. They think "Here is a debt—my sin, my guilt. This needs cancelling. The Atonement does this." So far well. But they forget "Here besides is a disease which requires rooting out, and being rooted out, a new nature, the very nature of Christ Himself, has to be established in the disciple." No amount of propitiation will effect this. It requires, first of all, the establishment of a vital connexion with Christ. This is the individual appropriation of the fruits of the Atonement.

